# Applications of gene pair methods in clinical research: advancing precision medicine

**DOI:** 10.1186/s43556-025-00263-w

**Published:** 2025-04-09

**Authors:** Changchun Wu, Xueqin Xie, Xin Yang, Mengze Du, Hao Lin, Jian Huang

**Affiliations:** 1 The Clinical Hospital of Chengdu Brain Science Institute, School of Life Science and Technology, University of Electronic Science and Technology of China, Chengdu, 611731 China; 2 School of Healthcare Technology, Chengdu Neusoft University, Chengdu, 611844 China

**Keywords:** Gene pair, High-throughput sequencing, Ranking relationships, Clinical research, Batch effects

## Abstract

The rapid evolution of high-throughput sequencing technologies has revolutionized biomedical research, producing vast amounts of gene expression data that hold immense potential for biological discovery and clinical applications. Effectively mining these large-scale, high-dimensional data is crucial for facilitating disease detection, subtype differentiation, and understanding the molecular mechanisms underlying disease progression. However, the conventional paradigm of single-gene profiling, measuring absolute expression levels of individual genes, faces critical limitations in clinical implementation. These include vulnerability to batch effects and platform-dependent normalization requirements. In contrast, emerging approaches analyzing relative expression relationships between gene pairs demonstrate unique advantages. By focusing on binary comparisons of two genes’ expression magnitudes, these methods inherently normalize experimental variations while capturing biologically stable interaction patterns. In this review, we systematically evaluate gene pair-based analytical frameworks. We classify eleven computational approaches into two fundamental categories: expression value-based methods quantifying differential expression patterns, and rank-based methods exploiting transcriptional ordering relationships. To bridge methodological development with practical implementation, we establish a reproducible analytical pipeline incorporating feature selection, classifier construction, and model evaluation modules using real-world benchmark datasets from pulmonary tuberculosis studies. These findings position gene pair analysis as a transformative paradigm for mining high-dimensional omics data, with direct implications for precision biomarker discovery and mechanistic studies of disease progression.

## Introduction

Genes are the fundamental units of life, playing a crucial role in the inheritance and functional expression within organisms. All life phenomena, such as birth, growth, illness, aging, and death, are related to gene [1–4]. High-throughput sequencing technology [5] and DNA microarray technology [6] are crucial tools in genomics research, enabling scientists to monitor gene expression on a genomic scale [7]. With the widespread application of these technologies, a large amount of high-throughput gene expression data from cancerous and non-cancerous tissue samples has been generated. These provide a wealth of information, although it is provided only implicitly in the form of raw expression values [8]. Effectively utilizing this information can have a significant impact on disease detection, subtype classification, prognosis, and disease progression [9].

In clinical research, differential expression analysis based on single-gene raw expression levels has been widely used [10–12]. Common differential expression analysis methods can be categorized into several types. First, early single-gene differential expression analysis methods are typically based on hypothesis testing, including t-test, Analysis of Variance (ANOVA), and non-parametric test [13–16]. These methods assess the significance of gene expression differences under different conditions using traditional statistical hypothesis testing, and are suitable for simpler experimental designs with small sample sizes. Second, the significance analysis of microarrays (SAM) is a statistical inference method that uses ranking-based hypothesis testing to evaluate differential expression between groups. Originally developed for microarray data, SAM is also widely used in RNA-seq analysis [17–19], particularly for controlling false positives in multiple hypothesis testing. Additionally, methods like limma (based on linear models and Bayesian methods) [20–22], edgeR [23–25], and DESeq2 [26–28] (which use negative binomial models) are widely used in RNA-seq analysis. These methods excel in handling complex experimental designs and multiple conditions, offering more comprehensive differential expression results. These representative single-gene differential expression analysis methods provide powerful tools for biological research and have uncovered many important biological insights across various fields. For example, Tabone et al. have developed an optimized and simplified feature to differentiate between active tuberculosis and other lung diseases with gene expression profiles [29]. Sun et al. revealed the potential of *CLEC3B* in lung cancer diagnosis [30]. Lee et al. identified a novel 5-gene biomarker for non-invasive diagnosis of gastric cancer, which may serve as a potential diagnostic tool for early detection [31]. Furthermore, researchers have identified numerous molecular markers associated with subtype classification [32, 33], disease prognosis [34, 35], and disease progression [36, 37]. These studies, although extracting molecular features related to specific issues from high-dimensional gene data, mainly focus on the level of individual genes. It is noteworthy that diseases are often result from interactions among multiple genes and other molecules within biological pathways or gene regulatory networks [38, 39]. Typically, genes within a network collaborate synergistically, and the effect of one gene can significantly influence the activity of other genes. As a result, insights from the expression patterns of individual genes are often limited [40].

**Fig. 1:**
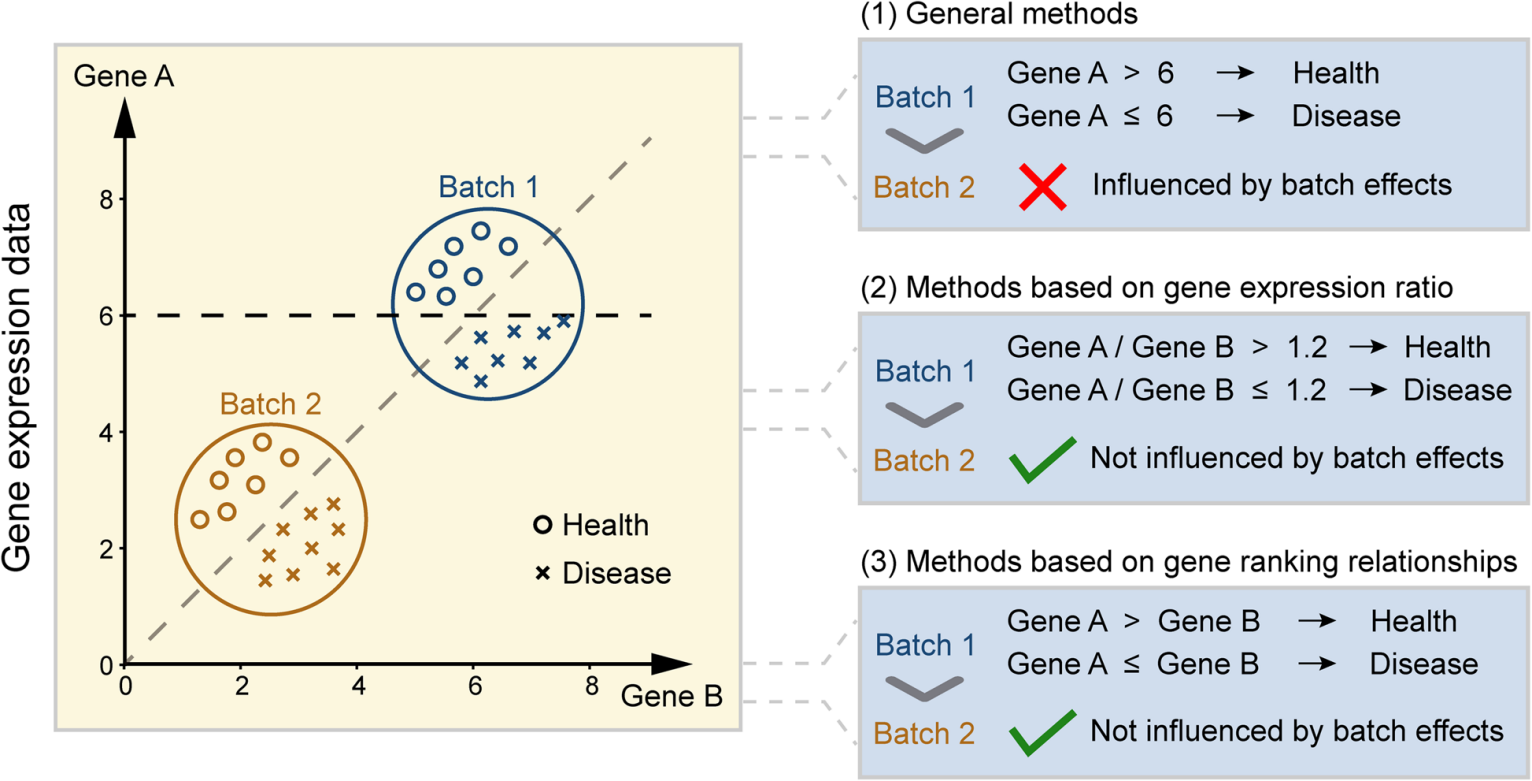
Illustration of batch effects in gene expression profiles. Gene expression profiles from two batches (blue and brown) were used to distinguish disease (cross) from healthy control (circle) samples. The X-axis and Y-axis represent the expression levels of genes B and A, respectively. In Batch 1, a fixed threshold for Gene A’s expression level (e.g., Gene A > 6) can effectively classify samples as healthy or diseased. However, this approach fails in Batch 2, highlighting the influence of batch effects when relying on absolute expression levels of specific genes. In contrast, methods based on relative changes between two genes, such as relative expression rankings or ratios, are robust to batch effects and yield consistent classification results across both batches. For instance, in both Batch 1 and Batch 2, a sample is classified as healthy if the expression value of Gene A is consistently higher than Gene B; otherwise, it is classified as diseased

As an alternative approach, assessing the relationships between a few genes, such as their interaction patterns, can provide more valuable information on disease-related biomolecular processes [41]. This concept was first put into practice in Bø’s study, which used gene pair features to distinguish between colorectal cancer and normal controls, showing a high level of diagnostic accuracy [42]. The emergence of gene pair method provided new perspectives for subsequent research, leading to the gradual development of many new gene pair-based methods, which have been widely applied and have become reliable sources for biological discoveries [43–47]. To date, these methods can be divided into two main categories. One category is based on gene expression values. These methods combine the expression values of two genes to create new features that can be applied to various clinical issues. Compared with single-gene expression patterns, these approaches may provide more useful insights. Moreover, some methods are robust to batch effects from different experimental platforms (Fig. 1). The other category focuses on gene ranking relationships. These methods depend solely on comparing the expression values of two genes, making them also unaffected by batch effects and eliminating the need for data normalization. As a result, they produce accurate, robust, and easily interpretable results with strong clinical practicality (Fig. 1).

As described above, gene pair methods were first introduced in 2002 [42]. Since then, many new approaches have been developed. In this review, we systematically assessed various gene pair-based data mining algorithms so as to provide insights and guidance for researchers to choose appropriate approaches for effective biomolecular explorations and clinical research. Based on existing methods, we primarily examine eleven gene pair-based approaches—three based on gene expression values and eight based on gene ranking relationships—that were developed using mRNA transcriptome data and binary classification problems. To further promote their application, we constructed a reproducible analytical pipeline based on published methodologies. Using real-world peripheral blood transcriptome gene expression profiles from pulmonary tuberculosis (PTB) as benchmark datasets, we replicated the various methods and evaluated their performance in addressing the biological challenge of PTB diagnosis. Notably, these gene pair-based methods can also be applied to other types and different omics data. This versatility allows them to better serve various clinical scenarios, such as diagnosis, prognosis, and disease progression, thereby advancing medical progress.

## Gene pair methods based on gene expression values

**Fig. 2:**
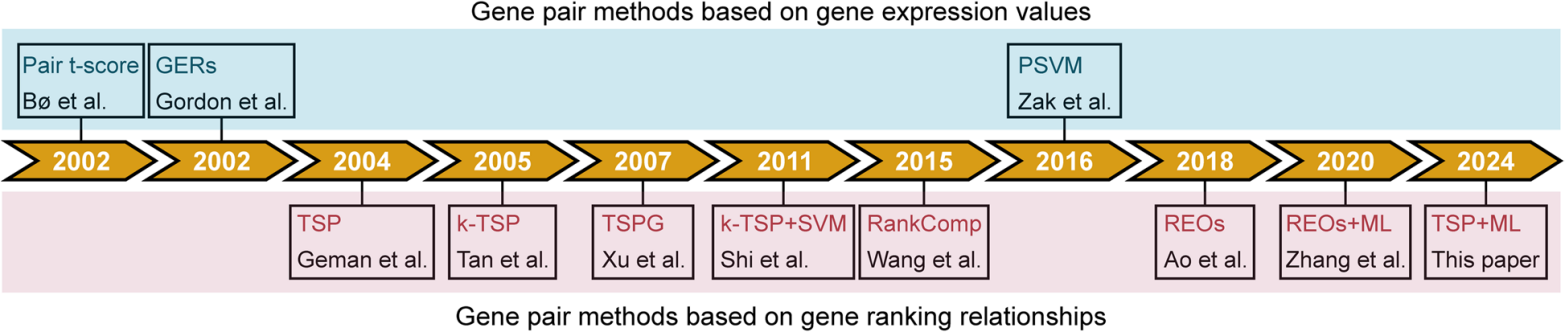
Timeline of the development of gene pair methods. All the gene pair methods were categorized based on their underlying principles: one group relies on gene expression values, while the other focuses on gene ranking relationships. Methods based on gene expression values (blue) include approaches such as Pair t-score, GERs, and PSVM. Methods based on gene ranking relationships (pink) encompass TSP, *k*-TSP, TSPG, and subsequent advancements like *k*-TSP + SVM, RankComp, REOs, REOs + ML and the integration of TSP with machine learning proposed in this study

There are three gene pair methods based on gene expression values: Pair t-score, Gene Expression Ratios (GERs), and Pair-wise Support Vector Machine ensembles (PSVM) (Fig. 2, Table 1). When detailing each method, unless otherwise specified, it is assumed that the columns of the gene expression profile used for training represent samples, while the rows represent genes.

**Table 1:** Gene pair methods based on gene expression values

Methods	Author(s)	Year	ML^a^	Application	Batch Effects^b^	Availability
Pair t-score [42]	Bø et al	2002	Yes	Classification Task	No	J-Express software
GERs [48]	Gordon et al	2002	No	Classification Task	Yes	No
PSVM [49]	Zak et al	2016	Yes	Classification Task	No	No

*Abbreviation:* *ML* Machine Learning

^a^Whether machine learning methods are used

^b^Whether it is not affected by batch effects

### Pair t-score

Pair t-score assigns a score for each gene pair based on their expression values, reflecting how effectively the pair distinguishes between two experimental classes. Proposed by Bø et al. in 2002, this method introduced the concept of gene pairs for the first time [42]. The specific description is as follows:

1. Projection onto the diagonal linear discriminant (DLD) axis [50]: Pairwise combinations of genes from the gene expression profile are used to form gene pairs. For each gene pair, project the gene expression data onto a DLD axis defined by these two genes;
2. Calculation of the t-score: For the projected data points, calculate the two-sample t-statistic [51] between the two experimental classes. This statistic is used as the gene pair score to measure the ability of the pair to distinguish between different experimental classes. Based on the scores, a feature subset is selected using one of two methods: an exhaustive method called “all pairs” or a faster method called “greedy pairs”;
3. All pairs: First, select the gene pair with the highest score, then remove all pairs containing either of these two genes from the list. Next, choose the highest-scoring pair from the remaining list. Repeat this process until the desired subset is obtained;
4. Greedy pairs: First, rank all genes based on their individual t-scores. Subsequently, this method selects the gene *G*_*i*_ ​with the highest t-score. Then, it identifies gene *G*_*j*​_ that together with *G*_*i*_ ​maximizes the pair t-score. These two genes are then removed from the gene set, and the process is repeated on the remaining gene set until the desired number of genes is selected;
5. Based on the final selected gene pair features, various machine learning algorithms can be combined and applied across multiple clinical scenarios.

The authors applied this method to two public datasets: one comprising samples from colon cancer and normal tissue [52], and the other containing samples from acute lymphoblastic leukemia (ALL) and acute myeloid leukemia (AML) [53]. They demonstrated that accurate diagnosis can be achieved using only 20–30 and 15–20 gene expression levels, respectively. By observing gene pairs, they found that some genes are not effective discriminators when used alone but perform well when paired with others, indicating that gene combinations reveal interesting information that cannot be discovered through other methods. The Pair t-score was integrated into the J-Express software package by the author [54]. This method has subsequently been applied and has provided new insights for many studies [55–58].

### Gene Expression Ratios

The method aims to accurately differentiate different experimental classes using gene expression ratios and reasonably chosen thresholds. It was proposed by Gordon et al. in 2002 [48]. The authors tested the accuracy of this method in distinguishing between malignant pleural mesothelioma (MPM) and adenocarcinoma (ADCA) in 181 tissue samples (31 MPM and 150 ADCA). They identified gene pairs with highly significant, negatively correlated expression levels using a training set of 32 samples (16 MPM and 16 ADCA), forming a total of 15 ratios from expression profile. Each ratio achieved a diagnostic accuracy of at least 90% in predicting the remaining 149 samples. They further tested the accuracy of multiple ratio combinations, achieving diagnostic accuracies of 95% for MPM versus ADCA using two ratios and 99% with three ratios. The detailed method is described below:

1. Identifying genes with significantly different average expression levels between two experimental categories based on gene expression profile;
2. Select two or more genes with the most significant differential expression, ensuring that these genes are not simultaneously highly expressed in one class relative to the other;
3. Select a class, then divide each gene with relatively high expression in this class (*N*) by each of the remaining genes (*M*) to obtain N*M expression ratios;
4. For a given expression ratio, if > 1 is classified as the selected class, < 1 is another class. Based on this rule, the sample category can be determined by a single expression ratio or by combining multiple expression ratios using a majority voting principle [59].

The GERs method is unaffected by data collection platforms and does not require correction for batch effects. The authors who proposed this method have shown its utility in predicting the efficacy of medulloblastoma treatment. Similarly, another study has shown that GERs can predict clinical outcomes in breast cancer (BC) patients treated with tamoxifen [60]. Furthermore, numerous studies have utilized this method, including in the diagnosis of head and neck squamous cell carcinoma [61] and gastric cancer [62], illustrating its applicability across various clinical scenarios.

### Pair-wise Support Vector Machine ensembles

The method combines gene pairs with a linear support vector machines algorithm (SVM), as proposed by Zak et al. in 2016 [49]. Using this method, they identified 16 risk gene features from 46 tuberculosis progressors and 107 matched controls. These features predicted tuberculosis progression with a sensitivity of 66.1% and a specificity of 80.6% in the 12 months preceding tuberculosis diagnosis. The detailed steps are as follows:

1. Based on gene expression profile data, randomly select 80% of the samples from each experimental category to perform differential analysis and identify differentially expressed genes;
2. Train SVM-based models separately on all pairwise combinations of the differentially expressed genes;
3. Using the remaining samples, set an accuracy threshold. Gene pairs that meet this threshold were given a score of “1”, while those that do not were given a score of “0”. Repeat the previous three steps fifty times. Finally, the gene pairs score between 0 and 50. Those with a score of 45 or higher are retained as the final feature set;
4. Construct an SVM model for each gene pair in the final feature set. Use the predictions from all SVM models and apply the majority voting principle to distinguish between the two experimental categories.

## Gene pair methods based on gene ranking relationships

There are eight gene pair methods based on gene ranking relationships, namely Top-Scoring Pair (TSP), *k*-Top Scoring Pairs (*k*-TSP), Top-Scoring Pair of Groups (TSPG), *k*-TSP + SVM, Rank Comparison (RankComp), Relative Expression Orderings (REOs), REOs + Machine Learning (REOs + ML), and TSP + Machine Learning (TSP + ML) (Fig. 2, Table 2). When introducing each method in detail, unless specifically stated otherwise, it is assumed that the columns of the gene expression profile used for training represent samples and the rows represent genes.

**Table 2:** Gene pair methods based on gene ranking relationships

Methods	Author(s)	Year	ML^a^	Application	Batch Effects^b^	Availability
TSP [63]	Geman et al	2004	No	Classification Task	Yes	R Package
*k*-TSP [64]	Tan et al	2005	No	Classification Task	Yes	R Package
TSPG [65]	Xu et al	2007	No	Classification Task	Yes	No
*k*-TSP + SVM [66]	Shi et al	2011	Yes	Classification Task	Yes	No
RankComp [67]	Wang et al	2015	No	Individualized DEA	Yes	R Package
REOs [68]	Ao et al	2018	No	Classification Task	Yes	No
REOs + ML [69]	Zhang et al	2020	Yes	Classification Task	Yes	No
TSP + ML	This article	2024	Yes	Classification Task	Yes	No

^a^Whether machine learning methods are used

^b^Whether it is not affected by batch effects

Abbreviation: ML, Machine Learning; DEA, differential expression analysis

### Top-Scoring Pair

This is a novel method for microarray data classification based on pairwise comparison of relative gene expression levels, proposed by Geman et al. in 2004 [63]. The authors demonstrated the effectiveness of this method in three classification problems: predicting lymph node status in BC patients, classifying leukemia subtypes, and distinguishing prostate cancer patients from normal controls [53, 70, 71]. This method specifically involves the following steps:

1. Assuming the columns of the gene expression profile represent samples (*X*_*1*_, …, *X*_*N*_) and rows represent genes (*G*_*1*_, …, *G*_*P*_), where the expression value of the *i*-th gene *G*_*i*_ in the *n*-th sample *X*_*n*_ is denoted as *X*_*i,n*_. For binary classification, set the vector of class labels for the samples be (*Y*_*1*_, …, *Y*_*N*_), where *Y*_*n*_ belongs to *C* = {*C*_*1*_, *C*_*2*_};
2. Within each sample, rank all genes based on their expression levels, replacing the expression values of each gene with its rank. This process results in a new matrix *R*, where the rank of the *i*-th gene *G*_*i*_ in the *n*-th sample *X*_*n*_ is denoted as *R*_*i,n*_;
3. Pair the genes to form gene pairs (*G*_*i*_, *G*_*j*_) where *i*, *j* belong to {*1*, …, *P*} and *i* ≠ *j*. For each gene pair (*G*_*i*_, *G*_*j*_), calculate the frequency *P*_*ij*_ of *R*_*i*_ < *R*_*j*_ in each of the two classes of samples;
  1 $${P}_{ij}\left({C}_{m}\right)=Prob\left({R}_{i}<{R}_{j} | Y={C}_{m}\right), m=\left\{1, 2\right\}$$
4. Here Δ_*ij*_ represents the "score" for the gene pair (*G*_*i*_, *G*_*j*_), and select the gene pair(s) with the highest score as the top-scoring pair(s);
  2 $$\triangle_{ij}\;=\;\left|P_{ij}\left(C_1\right)\;-\;P_{ij}\left(C_2\right)\right|$$
5. If the top-scoring pair is (*G*_*i*_, *G*_*j*_) and assuming *P*_*ij*_(*C*_*1*_) > *P*_*ij*_(*C*_*2*_), the new sample *X*_*new*_ is classified according to the following rule:
  3 $${Y}_{new}=\left\{\begin{array}{c}{C}_{1},\ if\ {R}_{i,\ new}<{R}_{j,new},\\ {C}_{2},\ Otherwise. \end{array}\right.$$
  If *P*_*ij*_(*C*_*2*_) > *P*_*ij*_(*C*_*1*_), the classification rule is reversed. If there are multiple top-scoring pairs, the final class is determined based on the majority voting principle using the above rules.

The motivation for proposing this method arises from the technical and practical limitations currently faced when using gene expression microarray data for class prediction, such as disease detection, tumor identification, and treatment response prediction. For instance, the number of observed samples is typically small, often only a few dozen, while the number of genes is very large, usually in the thousands, making accurate statistical inference challenging. Moreover, traditional machine learning methods are often difficult to interpret in simple or biologically meaningful terms [72, 73]. In contrast, the TSP method provides decision rules that (1) involve only a very small number of genes and their ranking relationships [74, 75], (2) are both accurate and interpretable [76, 77], and (3) achieve prediction rates for cancer data comparable to those in previous studies that used more genes and more complex procedures. The ‘tspair’ R package has been developed based on the TSP method [78]. This method has also been applied in various studies. Isella et al. utilized this approach to derive an algorithm for assigning colorectal cancer subtypes, termed CRIS-TSP, through which they identified five CRC subtypes with distinct molecular, functional, and phenotypic characteristics [79]. Hua et al. established a prognostic model for ovarian tumors by analyzing 1580 transcriptome profiles and combining the TSP method [80]. Zhao et al. used the method to predict which patients would experience systemic tumor progression following radical prostatectomy [81]. Weichselbaum et al. developed predictive biomarkers for chemotherapy and radiotherapy in BC using this method [82]. Raponi et al. applied this method to construct a predictor for the response of AML patients to the farnesyltransferase inhibitor tipifarnib [83]. Additionally, this method has provided new insights for many studies, such as the extraction of prognostic features for stage I non-small cell lung cancer (NSCLC) [84], and the development of the Top-Scoring Triplet (TST) classifier based on the expression orderings among three genes [85]; presents TimiGP, a novel gene-pair-based method for analyzing the tumor immune microenvironment [86]; developed ranktreeEnsemble by integrating TSP with boosting and random forest to enhance disease classification based on gene expression [87]; combines decision trees and evolutionary multi-test tree (EMTTree) with relative expression (RX) to develop Relative Multi-test Classification Tree (RMCT) [88] and EMTTree + RX [89], which enhances multi-omics data classification.

### *k*-Top Scoring Pairs

In certain situations, the TSP method may be affected when the training data is perturbed by adding or removing some samples. Therefore, in 2005, Tan et al. proposed the *k*-TSP method, which extends TSP and aims to address this issue [64]. The *k*-TSP method can be seen as an ensemble learning approach, classifying based on *k* disjoint top-scoring pairs. In other words, it leverages the discriminative power of multiple "weaker" rules to make more reliable predictions. This method employs straightforward and accurate decision rules, focusing on the ranking relationships of a limited number of gene pairs. The authors compared this approach to other machine learning techniques for class prediction in 19 binary and multi-class gene expression datasets involving human cancers (including colorectal cancer, leukemia, lung cancer, prostate cancer, etc.) [90–92]. The results showed that the *k*-TSP method performed as efficiently as SVM and outperformed other machine learning methods (Decision Trees [93], K-Nearest Neighbors [94], and Naive Bayes [95]). Here is a detailed description of the method:

1. According to the specific description (i-iv) of the TSP method as above, obtain the scores Δij for gene pairs and sort them in descending order;
2. Since multiple gene pairs may achieve the same highest score, calculate the secondary score Γ_*ij*_, known as the rank difference, to break ties. For these gene pairs, first compute the average rank difference γ_*ij*_ within each class of samples:
  4 $${\upgamma }_{ij}\left({C}_{m}\right)=\frac{{\sum }_{n\in {C}_{m}}\left({R}_{i,n}-{R}_{j,n}\right)}{\left|{C}_{m}\right|}, m=\left\{1, 2\right\}$$
  Where |*C*_*m*_| represents the number of samples in each different category. *R*_*i,n*_ and *R*_*j,n*_ are defined as described in the TSP method. Next, you can proceed to calculate Γ_*ij*_:
  5 $${\Gamma }_{ij}=\left|{\upgamma }_{ij}\left({C}_{1}\right)-{\upgamma }_{ij}\left({C}_{2}\right)\right|$$
  From the formula, it can be derived that Γ_*ij*_ represents the extent of reversal of the gene pair between the two classes of samples. Ultimately, for gene pairs where Δ_*ij*_ is equal, Γ_*ij*_ breaks the tie, resulting in a complete ranking of gene pairs in descending order;
3. First, select the first gene pair in the ranking. Then, remove all pairs from the ranking that include either of these two genes. Next, select the first pair from the remaining ranking. Repeat this process until *k* disjoint highest-scoring pairs are selected. The parameter *k* is determined through cross-validation and must be odd to break ties in majority voting procedures;
4. Finally, for each of the *k* pairs of genes, vote according to formula (3) and determine the class of the new sample based on the majority voting principle.

This method typically involves fewer genes, providing biologically meaningful decision rules, and thus has widespread application. For instance, it has been applied in studies investigating the anti-tumor activity of AL101 against adenoid cystic carcinoma with activated NOTCH signaling [96], constructing a classifier for predicting prostate cancer progression [97], developing personalized immune prognostic features for early-stage nonsquamous NSCLC [98], developing a model to predict the likelihood of hepatocellular carcinoma (HCC) recurrence after liver transplantation [99], and building highly accurate classifiers to differentiate gastrointestinal stromal tumors from leiomyosarcomas [100]. These application examples, which involve clinical issues such as diagnosis, prognosis assessment, and progression risk, demonstrate the robustness of the *k*-TSP method. Furthermore, other studies have developed R packages for this method. For example, the ‘ktspair’ R package (already disappeared from C-RAN), was developed by Damond et al. in 2011 [101]. Additionally, Afsari et al. developed the ‘switchBox’ R package in 2015, which is also based on the *k*-TSP method and has been widely applied [102–105]. This package employs a new approach, namely “ANOVA”, to select the number of pairs (*k*). Compared to the original method, this approach is less computationally intensive and less prone to overfitting [106].

### Top-Scoring Pair of Groups

To identify common gene expression features across various types of cancer, Xu et al. proposed the TSPG method in 2007. This method retains the basic properties of the TSP classifier but introduces a repeated random sampling strategy, incorporating more genes into the decision-making process [65]. The motivation for developing this method is as follows: First, cancer results from the combined action of multiple genes. To better understand the mechanisms of cancer development, it is necessary to identify common cancer features composed of multiple genes; Additionally, in some cases, when the training data is perturbed by adding or removing samples, a gene may form the top-scoring pair with different genes. This indicates that genes consistently appearing in top-scoring pairs are likely closely related to cancer, while other genes paired occasionally might not be. To retain genes that play critical roles in the carcinogenesis of common cancers and eliminate those possibly unrelated to cancer, a repeated random sampling strategy is required [107]. In their study, the authors collected and integrated approximately 1500 microarray gene expression profiles from 26 published cancer datasets, involving 21 major human cancer types (including lung, breast, bladder, ovary, pancreas, etc.) [108–110]. They then applied TSPG to the training datasets and identified a common cancer feature composed of 46 genes. After further validation with training and independent test datasets, this feature was found to be potentially useful as a robust and objective cancer diagnostic test. Here are the ways in detail:

1. Randomly select *S*% (in this study, *S* = 90) of the total samples from the gene expression profile used for training;
2. Obtain the complete ranking of gene pairs from highest to lowest according to the specific description (i-ii) of the *k*-TSP method;
3. First, select the top gene pair from the ranking and remove all pairs from the ranking that include either of these two genes. Next, select the top pair from the remaining ranking. Repeat this process until you have selected *k* (in this study, *k* = 20) disjoint highest-scoring pairs. Then, for each of these *k* gene pairs (*G*_*i*_, *G*_*j*_), if *P*_*ij*_(*C*_*1*_) > *P*_*ij*_(*C*_*2*_) (The definition of *P*_*ij*_ as described in the TSP method), assign *G*_*i*_ to Group 1 and *G*_*j*_ to Group 2; otherwise, assign *G*_*j*_ to Group 1 and *G*_*i*_ to Group 2. Genes assigned to Group 1 typically exhibit lower expression levels compared to those assigned to Group 2;
4. Repeat the preceding steps (i-iii) *N* times (in this study, *N* = 1000), after which the frequency of each gene in Group l and Group 2 is calculated separately. Set a frequency threshold *F* (in this study, *F* = 80%) to retain genes that appear more than this threshold;
5. For a new sample, rank all genes in Group 1 and Group 2 based on their expression levels. If the average ranking of genes in Group 1 is lower than that in Group 2, classify the sample as Class 1; otherwise, classify it as Class 2.

This method introduces repeated random sampling, which allows for the extraction of more robust and reliable features.

### *k*-Top Scoring Pairs + Support Vector Machine

The widely used *k*-TSP method is a simple yet powerful non-parametric classifier. However, its overall robustness may not extend to challenging datasets, possibly due to the relatively straightforward voting scheme used by the classifier. Therefore, Shi et al. (2011) proposed using multiple gene pairs obtained from *k*-TSP as features to construct a SVM-based model [66]. This approach was applied to four cancer prognosis datasets, where *k*-TSP + SVM outperformed the *k*-TSP classifier across all datasets, achieving performance comparable to or better than SVM used alone. Furthermore, this method has also been widely applied, including the construction of model for predicting melanoma subtypes [111].

### Rank Comparison

The differential expression analysis that focuses on inter-group comparisons can only capture differentially expressed genes at the population level, potentially masking the heterogeneity of individual differences [67]. Therefore, to provide patient-specific information for personalized medicine, it is necessary to conduct differential expression analysis at the individual level. Consequently, Wang et al. (2015) proposed a method to detect differentially expressed genes in individual disease samples by utilizing disrupted rankings within individual disease samples [112]. The principle is to use previously accumulated data to pre-determine the stable gene pairs in specific types of normal tissues. Based on these stable gene pairs, any disrupted genes and pathways in disease samples can be easily detected. In both simulated data and real paired cancer-normal sample data, this method demonstrated excellent performance. The identification and application of lung cancer prognostic biomarkers further proved the usefulness of the RankComp method in clinical practice [113, 114]. The specific description of the method is as follows:

1. Collect normal samples of a specific tissue type from various data sources. For each sample, convert the gene expression values to their ranks within the sample. Perform pairwise comparisons for all genes. For a gene pair (*G*_*i*_, *G*_*j*_), if the frequency of *G*_*i*_ > *G*_*j*_ or *G*_*i*_ < *G*_*j*_ in the normal samples is greater than 0.99, the pair is defined as a stable gene pair. Finally, take the intersection of genes from all identified stable gene pairs to obtain the gene list *G*;
2. For each gene *G*_*i*_​ in the gene list *G*, *G-pair* denote the set of stable gene pairs in normal samples that include *G*_*i*_. Let *a* and *b* denote the numbers of gene pairs belonging to *G-pair* with ranking patterns *G*_*i*_ > *G*_*j*_ and *G*_*i*_ < *G*_*j’*_ in normal samples, and *c* and *d* denote the corresponding numbers of gene pairs belonging to *G-pair* with ordering patterns *G*_*i*_ > *G*_*j*_ and *G*_*i*_ < *G*_*j’*_ in the disease sample *k*. Use Fisher’s exact test under the null hypothesis that *a*/*b* = *c*/*d* to determine whether *G*_*i*_ is differentially expressed in disease sample *k*. If there is significant evidence to reject the null hypothesis, then *G*_*i*_ ​is considered differentially expressed. If *a*/*b* < *c*/*d*, *G*_*i*_ ​is defined as upregulated; if *a*/*b* > *c*/*d*, *Gi* is defined as downregulated;
3. For each disease sample, iterate through each gene in the gene list *G* following the procedure in step ii to determine which genes are differentially expressed in the corresponding disease sample.

The analysis of individual-level differential genes has significant applications. It enables pathway analysis at the individual level. Additionally, it allows for patient stratification directly based on the specific dysregulation status of each patient. This method has also provided new insights and approaches for many other studies. For example, Song et al. identified a seven autophagy related gene pairs signature using the RankComp algorithm for colorectal cancer diagnosis, which could distinguish cancerous from non-cancerous tissues with high accuracy and was validated across multiple datasets [115]. Hu et al. used this method to identify differentially expressed genes in normal and osteosarcoma tissue samples [116]. Cai et al. developed RankCompV2 based on this method [117]. Moreover, Wang et al. proposed an approach to detect pathways with disrupted relative expression order at the individual level [118].

### Relative Expression Orderings

This method, which determines features for clinical application based on the relative expression order of gene pairs within samples, was proposed by Ao et al. in 2018 [68]. In their study, the authors used this method to identify a signature of 19 gene pairs from training datasets of hepatocellular carcinoma (HCC) to distinguish HCC and most of tumor-adjacent tissues from cirrhosis tissues of non-HCC patients. These signatures were validated in two large sample sets from biopsy and surgical resection specimens. The results indicate that even if the biopsy sample is not taken from an accurate location, this feature can still effectively distinguish different samples, demonstrating the practicality of this method. The specific steps of this method are as follows:

1. For each gene pair (*G*_*i*_, *G*_*j*_), if the REO pattern (*G*_*i*_ > *G*_*j*_ or *G*_*i*_ < *G*_*j*_) remains consistent across more than 85% of the samples in the first class in the training data, and reverses in more than 85% of the samples in the second class, then this gene pair is defined as a reverse gene pair between these two types of samples. The calculation of the ranking difference for reverse gene pairs in each sample is as follows:
  6 $${R}_{ij}=\left|{R}_{i}-{R}_{j}\right|$$
  where *R*_*i*_ and *R*_*j*_ represent the rankings of genes *G*_*i*_ and *G*_*j*_ in the sample respectively, *R*_*ij*_ denotes the absolute rank difference between the two genes;
2. The mean[*R*_*ij*_(*C*_*1*_)] and mean[*R*_*ij*_(*C*_*2*_)] represent the mean absolute rank differences of reversed gene pairs (*G*_*i*_, *G*_*j*_) in all samples of the first and second classes, respectively. Then, calculate the geometric mean of the mean[*R*_*ij*_(*C*_*1*_)] and mean[*R*_*ij*_(*C*_*2*_)] to assess the degree of reversal of this gene pair between the two classes of samples:
  7 $$avg{R}_{ij}=\sqrt{mean\left[{R}_{ij}\left({C}_{1}\right)\right]\times mean\left[{R}_{ij}\left({C}_{2}\right)\right]}$$
  The larger this geometric mean, the greater the degree of reversal of this gene pair between the two types of samples;
3. All gene pairs are sorted in descending order based on their degree of reversal. The gene pair with the maximum degree of reversal is selected as the seed, and then a forward selection procedure [119] is used to search for the optimal subset of an odd number of gene pairs;
4. Based on the subset of reversed gene pairs, classification for a given sample is achieved using a majority voting rule. Specifically, if more than half of the feature gene pairs exhibit the REO pattern of the first class, the sample is classified into that class; otherwise, it is classified into the second class.

This method has been adopted in numerous studies. For instance, it has been applied in the diagnosis of pancreatic cancer [120], colorectal cancer [121], gastric cancer [122], as well as in prognostic prediction for hepatocellular carcinoma [123, 124], and many others.

### Relative Expression Orderings + Machine Learning

The method was proposed by Zhang et al. in 2020, combining Machine Learning (ML) methods with REOs [69]. In the study, the authors initially extracted gene pair features using REOs from the HCC gene expression profile. They then applied Maximum Relevance Minimum Redundancy (mRMR) [125] and Incremental Feature Selection (IFS) [126] to eliminate irrelevant features, resulting in the identification of 11 gene pairs. These pairs were integrated with SVM to establish a diagnostic model for HCC. The authors further validated its ability to distinguish HCC using several independent datasets. The results indicate that the model can differentiate HCC from non-cancerous liver tissues and that these features exhibit robustness across clinical and pathological variations. The specific methodology is outlined as follows:

1. According to the specific description (i) of the REOs method, obtain the reverse gene pairs as preliminary features (with a threshold of 95% in this study);
2. Based on the gene expression profile and reversed gene pairs, a new matrix is generated with columns representing reversed gene pairs and rows representing samples. Each cell in the matrix is assigned a value to denote the relationship between the genes: 1 indicates *G*_*i*_ > *G*_*j*_, 0 indicates *G*_*i*_ < *G*_*j*_, − 1 indicates other situations, such as when *G*_*i*_ or *G*_*j*_ expression data is unavailable or undefined;
3. Based on the new matrix, mRMR is applied to rank gene pairs according to their maximum relevance to disease type and minimum redundancy with other gene pairs. Subsequently, IFS is used to select the optimal gene pairs as final features;
4. An SVM-based model is established based on the final selected features.

This method has also been widely applied. For example, Xie et al. used it for type 2 diabetes diagnosis and achieved promising predictive performance in both training and external independent test datasets [127]. Zhang et al. identified 10 reversal differential partial correlation (RDC) gene pairs through reversal gene pair and differential partial correlation analyses, and then construct a machine learning model for pancreatic ductal adenocarcinoma (PDAC) recognition, achieving 96.1% accuracy in cross validation and outperforming gene expression based models [128].

### Top-Scoring Pair + Machine Learning

Based on the methods described above, we have found that features extracted from the order relationships of gene pairs can be effectively applied to various clinical scenarios. Therefore, drawing from this experience, we have also developed a method that combines TSP with ML methods. The specific method is as follows:

1. Gene Pair Scoring: Obtain the gene pair scores Δ_*ij*_ ​based on the specific description (i-iv) of the TSP method, and sort them in descending order;
2. Preliminary Feature Extraction: The Δ_*ij*_ scores range from 0 to 1. By setting a threshold (e.g., 0.5, depending on the situation), obtain preliminary features with scores greater than the threshold. If the same gene appears in different gene pairs, only the gene pair with the highest score is retained;
3. Feature Encoding: Encode the training set based on the extracted features. Use the format ‘*G*_*i*_ | *G*_*j*_’ as column names, and sample names as row names. The values in the matrix will be 1 (*G*_*i*_ < *G*_*j*_), 0 (*G*_*i*_ = *G*_*j*_), or −1 (*G*_*i*_ > *G*_*j*_);
4. Feature Importance Ranking: Based on the feature-encoded matrix, apply various feature importance ranking methods, such as Maximal Information Coefficient (MIC) [129], Chi-squared Test (Chi2) [130], mRMR, and Random Forest (RF) [131], to rank the preliminary features;
5. Feature Selection and Model Construction: Apply the IFS method for feature selection. This involves adding features step-by-step based on their importance rankings from each feature importance method (such as MIC, Chi2, mRMR, RF) into multiple algorithms including eXtreme Gradient Boosting (XGBoost), Logistic Regression (LR) [132], RF, and SVM. After adding each new feature, perform cross-validation [133] and grid search. Ultimately, select the best combination of features and algorithms based on the receiver operating characteristic (ROC) area under the curve (AUC). Finally, use the entire training set to construct the final model.

After obtaining initial features through TSP, this method no longer relies on simple scoring for feature selection. Instead, it applies various feature importance ranking and IFS methods. This approach enables a more effective selection of features that are more relevant to the research objectives. Furthermore, comparing multiple algorithms ultimately yields the optimal model tailored for specific clinical issues.

## Comparison of gene pair methods

Among the eleven gene pair methods, all except RankComp are applicable to classification task. However, the Pair t-score and PSVM methods are susceptible to batch effects. Therefore, we selected the remaining eight methods for comparison, comprising one method based on expression values and seven based on ranking relationships. These methods were implemented using Python (version 3.10.13) and R (version 4.2.3). Specifically, the *k*-TSP method was implemented with the switchBox package (version 1.34.0), while all other methods were implemented strictly according to their original descriptions. For machine learning components, we mainly utilized the scikit-learn package (version 1.3.2) [134] and the xgboost package (version 2.0.3). The complete code is publicly available (10.5281/zenodo.14948408).

To quantitatively compare and evaluate these eight methods, as well as to assess their applicability to real-world clinical problems, we collected four PTB-related datasets (GSE19439, GSE19442, GSE19444, and GSE83456) from the Gene Expression Omnibus (GEO) [135] database as benchmark datasets (*n* = 253) [136, 137]. The benchmark dataset includes 99 samples from PTB patients (positive group) and 154 samples from latent tuberculosis infection or healthy controls (negative group). PTB was confirmed by sputum culture [138], while latent tuberculosis infection was diagnosed using the Tuberculin Skin Test (TST) and Interferon-Gamma Release Assay (IGRA) [139–141]. The raw, non-normalized data from these datasets were first processed using the limma package (version 3.54.0) for background correction and normalization [142]. Subsequently, probe-to-gene symbol conversion was performed, and the intersecting genes across the four datasets were extracted. Finally, the first three datasets were used as discovery data, split into training and testing sets in a 7:3 ratio, while the fourth dataset was used as an external independent validation set (Table 3).

**Table 3:** Pulmonary tuberculosis benchmark datasets

	GEO accession	Country	PTB	LTBI	HC
Discovery data	GSE19439 [136]	England	13	17	12
	GSE19442 [136]	South Africa	20	31	
	GSE19444 [136]	England	21	21	12
Validation data	GSE83456 [137]	England	45		61

Abbreviation: PTB, Pulmonary Tuberculosis; LTBI, Latent Tuberculosis Infection; HC, Healthy Control

**Fig. 3:**
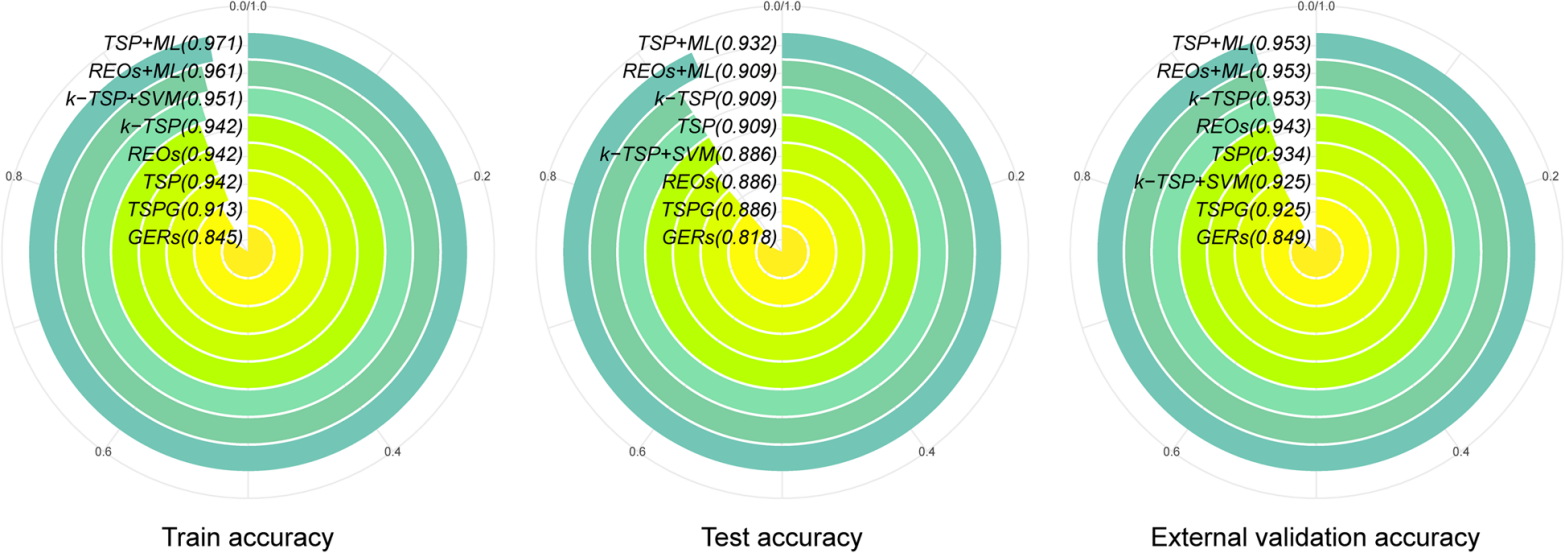
Performance comparison of gene pair methods. The selected eight methods were applied to the training set, testing set and external verification set of PTB benchmark data, and the performance was compared by accuracy

In the training set, we trained models using each of the eight methods and evaluated their performance on the training, testing, and external validation sets using accuracy as the metric (Fig. 3). From the perspective of overall performance, despite PTB being a non-cancer disease, these methods achieved an accuracy of 0.849–0.953 on the external validation set, demonstrating their potential for clinical applications. From the perspective of individual method performance, TSP combined with ML exhibited the best performance across the training, testing, and external validation sets. In terms of the number of feature genes used, these methods relied on only 2–30 genes to achieve excellent predictive performance. Notably, five genes—*ANKRD22*, *PSTPIP2*, *FCGR1BP*, *GBP6*, and *KLF12*—were selected as feature genes by more than half of the methods. Among them, *ANKRD22* has been confirmed as a PTB biomarker in multiple studies [143–145]. *GBP6*, a member of the guanylate-binding protein family, plays a critical role in innate immunity, and several studies have reported its association with PTB and can be used as a biomarker [146–148]. Although the other three genes have not been explicitly reported as PTB biomarkers, they are closely associated with the disease. For instance, *PSTPIP2* has been reported in studies related to PTB [29]. While *FCGR1BP* has not been directly identified as a PTB biomarker, its related genes, *FCGR1A* [149–152], *FCGR1B* [153, 154], and *FCGR1C* [155], have all been shown to be associated with PTB. *KLF12*, a transcription factor belonging to the family of Kruppel-like factors, whose members have been shown to play a key role in regulating the function of macrophages and T cells [156–158], and therefore it may be involved in the host immune response to Mycobacterium tuberculosis. In conclusion, gene pair methods not only identify known disease-associated genes but also uncover novel insights, providing valuable implications for addressing clinical challenges.

## Application of gene pair methods

**Fig. 4:**
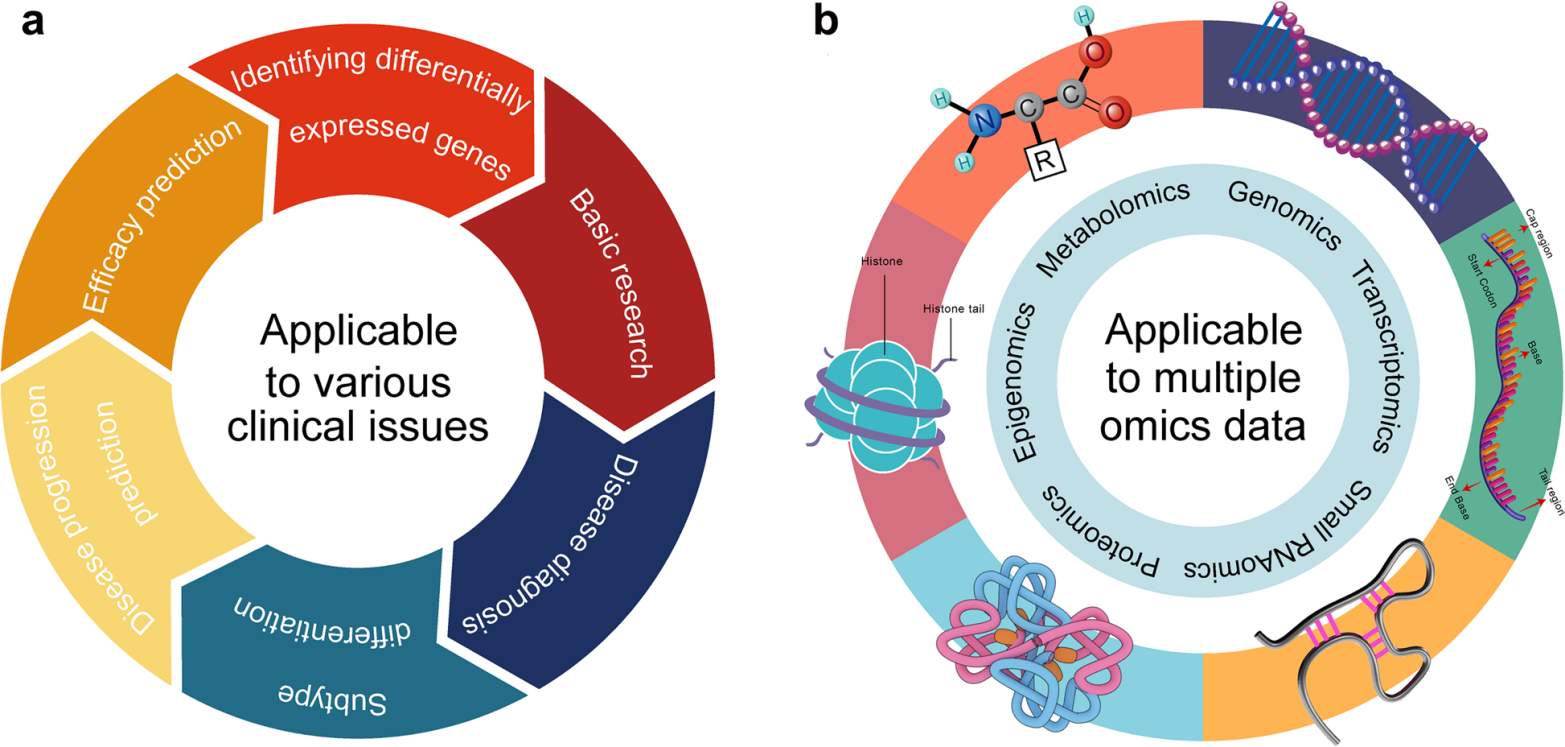
The diverse applications of gene pair methods across omics data and clinical issues. **a** In clinical contexts, gene pair methods demonstrate utility in addressing various issues, including identifying differentially expressed genes for basic research, diagnosing diseases, differentiating disease subtypes, predicting disease progression, and assessing therapeutic efficacy. Their robustness and flexibility enable seamless integration into clinical workflows for precision medicine and biomarker discovery. **b** Gene pair methods are versatile analytical tools that extend beyond transcriptomics to various omics layers, including metabolomics, genomics, epigenomics, proteomics, and small RNA. These methods utilize relationships between gene pairs to extract meaningful insights from high-dimensional data without requiring strict normalization, making them applicable to a wide range of omics datasets

Gene pair methods have been widely applied in research on both cancerous and non-cancerous diseases, encompassing various domains such as disease diagnosis, subtype classification, disease progression, and prognostic prediction (Fig. 4a). Here, we highlight several related studies that not only demonstrate the broad applicability of gene pair methods across diverse research scenarios but also underscore their excellent performance advantages, demonstrating their powerful potential in addressing complex biological challenges.

### Application in disease diagnosis

Pathologists face significant challenges in diagnosing pancreatic cancer (PC) using biopsy specimens, as these samples may be obtained from the wrong site or contain insufficient tissue [159]. Consequently, there is an urgent need to develop a platform-independent molecular classifier to accurately distinguish between benign pancreatic lesions and PC. To address this, Zhou et al. [120] utilized the REOs method to develop a robust qualitative mRNA signature capable of differentiating both PC tissues and cancer-adjacent normal tissues from non-PC pancreatitis and healthy pancreatic tissues. The training cohort included samples from five microarray datasets, comprising 74 normal pancreatic tissue, 72 pancreatitis tissue, and 269 PC tissue samples [160–163]. Using the training data, the authors constructed a feature set consisting of 12 gene pairs and 17 individual genes. The performance of this feature set was evaluated in an external validation cohort, which included 1,007 PC tissue samples and 257 non-tumor samples derived from both microarray and RNA-sequencing data [164–169]. Validation results demonstrated a geometric mean sensitivity and specificity of 96.7%, with an AUC of 0.978. Moreover, in 20 specimens obtained via endoscopic biopsy, the diagnostic accuracy of this feature set reached 100%. In summary, the REOs-based feature provides a reliable molecular diagnostic tool for PC, enabling objective differentiation between benign and malignant pancreatic lesions with high accuracy.

Xu et al. [170] utilized the TSP method to integrate microarray datasets from three independent prostate cancer (PCa) studies [71, 171, 172], identifying a robust gene pair (*HPN* and *STAT6*) for the diagnosis of PCa. The overall accuracy of this feature gene pair in independent datasets generated from different microarray platforms was 93.8%, with a sensitivity of 91.7% and specificity of 97.7% [173, 174].

With increasing research into the role of microRNAs (miRNAs) in cancer and their potential as diagnostic and prognostic biomarkers [175–177], Michele et al. [61] aimed to identify dysregulated miRNAs in head and neck squamous cell carcinoma (HNSCC) and evaluate their predictive capacity for the disease. RNA isolated from primary tumor tissues and non-diseased head and neck epithelial tissues, followed by microarray analysis to assess the expression of 662 miRNAs. Subsequently, miRNAs showing differential expression in both microarray and qRT-PCR analyses were further validated. The results identified 18 miRNAs with significantly altered expression between normal and tumor tissues. Notably, a biomarker based on the expression ratio of *miR-221*:*miR-375* demonstrated excellent predictive performance, achieving a sensitivity of 0.92 and a specificity of 0.93. These findings suggest that this biomarker holds promise as a potential high-value diagnostic tool.

Gastrointestinal stromal tumor (GIST) has emerged as a clinically distinct type of sarcoma, characterized by the frequent overexpression and mutation of the *c-Kit* oncogene and a favorable response to imatinib mesylate therapy [178]. However, distinguishing GIST from leiomyosarcomas (LMS) remains a significant diagnostic challenge [179, 180]. To improve diagnostic accuracy, Nathan et al. [100] conducted a genome-wide gene expression analysis of 68 tumor samples. Using the TSP method, the authors identified a single gene-pair classifier (*OBSCN* and *C9orf65*) capable of differentiating GIST from LMS. Validated this classifier by using RT-PCR on 20 samples in the microarray study and on an additional 19 independent samples, with 100% accuracy. This gene pair provides a rapid, PCR-based assay that reliably distinguishes GIST from LMS and has the potential to aid in diagnosis and in the selection of appropriate therapies.

Apart from the above examples, gene pair methods have also been used for the diagnosis of ischemic stroke [181].

### Application in subtype classification

Claudi et al. [79] deployed human-specific expression profiling of colorectal cancer (CRC) patient-derived xenografts (PDXs) to assess cancer-cell intrinsic transcriptional features. Through this approach, they identified five CRC intrinsic subtypes (CRIS) endowed with distinctive molecular, functional, and phenotypic characteristics. To translate the CRIS taxonomy into a clinically applicable diagnostic tool, the authors selected 526 shared CRIS genes from independent datasets across three different platforms (totaling 624 samples). Using the TSP and *k*-TSP methods, they developed a subtype assignment algorithm named CRIS-TSP. The results showed that the CRIS-TSP algorithm, using 40 gene pairs, maintained the classification ability of the original 526-gene classifier. When evaluated on six gene expression datasets with clinical outcome annotations (totaling 1,487 samples), the algorithm’s classification results further confirmed that CRIS-B subtype patients had a poorer prognosis. This suggests that the CRIS-TSP algorithm not only demonstrates high reliability in subtype classification but also holds potential clinical application value, offering guidance for personalized treatment in CRC patients.

Jennifer et al. [111], through gene expression analysis of 53 human melanoma cell lines and patient tumors, revealed that melanoma follows a two-dimensional differentiation trajectory, which can be further subdivided into four progressive subtypes. To train the subtype prediction model and enable more convenient cross-platform and cross-batch applications, the authors ultimately employed the TSP combined with the SVM method. Specifically, the authors selected the top 250 genes with the highest variance from the training set to construct the model, and converted the gene expression matrix into a binary matrix of gene pairs. Pairs were then scored by hypergeometric test to calculate the *p* value of enrichment for each subtype compared to the remaining subtypes. Gene pairs were filtered by a minimum *p* value of 1e-05 in at least one subtype, resulting in 1,561 gene pairs. Based on these selected gene pairs, a binary matrix for each cell line was constructed, and with the identified subtype information, an SVM model was trained using the radial basis function (RBF) kernel [182] from the R package kernlab. The model achieved a classification accuracy of 94% in leave-one-out cross-validation. This melanoma differentiation subtype prediction model can effectively support subtype diagnosis and assist in guiding personalized treatment strategies.

### Application in disease progression

To predict metastatic progression in PCa, Hubert et al. [97] collected and organized gene expression profiles from different datasets, including 1,239 primary tumor samples from PCa patients, with information on metastatic events. Each dataset was preprocessed, retaining only the common genes (12,761 genes) across all datasets. The data was then split into 75% training (*n* = 930) and 25% testing (*n* = 309) using stratified sampling [183]. Subsequently, a classifier was trained based on the training set using the *k*-TSP method, with features consisting of 13 up- and down-regulated gene pairs. In addition to its interpretable decision rules, this signature demonstrated robustness and stability in both the training and testing sets, with AUC values of 0.69 and 0.70, respectively. Moreover, the prognostic value of this feature was tested in an independent cohort of 439 primary tumor samples from PCa patients [184, 185], where the feature was found to be significantly associated with progression-free survival (PFS).

### Application in disease prognosis

Rajeshkumar et al. [186] aimed to evaluate the efficacy of AZD0530 (an orally active small molecule Src inhibitor) in a human PC xenograft and identify biomarkers that could predict its activity. The authors first treated 16 patient-derived PC xenografts with AZD0530 for 28 days. They then performed gene expression profiling on 16 tumor samples using microarrays, identified differentially expressed genes, and used the *k*-TSP method to identify a gene pair (*LRRC19* and *IGFBP2*) from the 16 training samples. This gene pair was used to build a classifier. In an independent test set comprising 8 xenografts, the classifier achieved a sensitivity of 100% and specificity of 83.3%. These results indicate that the gene pair has high predictive ability and could serve as a biomarker for PC sensitivity to AZD0530.

A study by Chen et al. [187] confirmed the effectiveness of the *GMPS*|*RAMP3* gene pair as a prognostic biomarker in HCC. By utilizing relative gene expression levels, the *GMPS*|*RAMP3* signature overcomes platform-specific biases and biological heterogeneity, ensuring robust prognostic predictions. Experimental validation further supported the role of *GMPS* in HCC, with *GMPS* knockdown suppressing proliferation, promoting apoptosis, and increasing gemcitabine sensitivity. These findings highlight the potential of gene pair-based strategies in prognostic assessment and personalized treatment approaches for HCC. Xu et al. [188] identifies a neutrophil extracellular traps (NETs)—related gene pair signature that can predict HCC prognosis and distinguish patient immune statuses, offering promise for immunotherapy strategies. In addition, Huang et al. [189] found that the *CSTF2*/*PDE2A* gene pair can predict the prognosis of HCC and regulate the cell cycle, showing promise as a novel prognostic predictor.

Most BC patients express estrogen receptor (ER) [190] and primarily receive tamoxifen as adjuvant therapy. However, approximately 40% of ER-positive BC patients either do not respond to tamoxifen or eventually develop resistance, leading to disease progression [191, 192]. Currently, clinical pathological features, such as tumor stage and grade, as well as the expression of *ERBB2* and *EGFR*, cannot accurately predict the clinical outcomes of tamoxifen treatment [193, 194]. Therefore, there is an urgent need to identify reliable predictive biomarkers. Ma et al. [60] analyzed the gene expression profiles of 60 ER-positive primary BC patients who received tamoxifen monotherapy as adjuvant treatment. The study found that the simple gene expression ratio of *HOXB13* to *IL17BR* could accurately predict the efficacy of tamoxifen treatment, showing superior predictive ability compared to existing biomarkers. In the tissue section dataset, the AUC value of this gene expression ratio reached 0.81; in the laser capture microdissection dataset, the AUC value further increased to 0.84. Moreover, overexpression of *HOXB13* was significantly associated with poor clinical prognosis and was closely related to the enhanced invasive ability of BC cells in vitro, suggesting that *HOXB13* may play a key role in BC progression [195]. To further validate their findings, the authors conducted an analysis on an independent cohort comprising formalin-fixed paraffin-embedded (FFPE) samples from 20 patients with ER-positive early primary BC who were treated with tamoxifen monotherapy. The results showed that the *HOXB13*:*IL17BR* expression ratio was significantly associated with the patients’ clinical outcome (t test *p* = 0.024), with higher *HOXB13* expression correlated with poor outcome. In conclusion, the *HOXB13*:*IL17BR* expression ratio has the potential to be a precise biomarker for predicting the efficacy of tamoxifen monotherapy, providing an important basis for personalized BC treatment.

Apart from the examples mentioned above, gene pair methods have also been used to develop prognostic models for various cancers, including cervical cancer [196], colon adenocarcinoma [197], gastric cancer [198], clear cell renal cell carcinoma [199], lung adenocarcinoma [200, 201], colorectal cancer [202, 203], and glioma [204, 205]. These methods have achieved significant results in predicting patient survival, risk stratification, and guiding treatment strategies.

## Extension of gene pair methods

### Extension to other data types

In this review, we primarily explore gene pair methods developed based on mRNA transcriptome data. However, these methods and their underlying principles are also applicable to other types of data (Fig. 4b). For example, Ren et al. used the GERs method to analyze microRNA expression data in osteosarcoma, developing biomarkers for predicting osteosarcoma [206]. Patnaik et al. utilized the TSP method to identify microRNA prognostic markers that predict the recurrence of NSCLC from microRNA expression data [207]. Lin et al. applied the *k*-TSP’s principle to serum metabolomics data obtained from liquid chromatography-mass spectrometry (LC–MS) of hepatocellular carcinoma (HCC) and chronic liver disease (CLD), ultimately selecting 27 feature pairs that successfully distinguished HCC from CLD and revealed certain deep metabolic disorders associated with liver disease progression [208]. Additionally, Yan et al. employed RankComp’s principle to accurately detect Differentially Methylated CpG sites in individual cancer samples from DNA methylation data of lung adenocarcinoma samples [209].

In addition to gene pair methods developed based on mRNA transcriptome data, there are also many gene pair methods designed for other types of data, and the concept of feature pairs has similarly been applied to non-gene expression data. For instance, gene pair methods have been developed for microRNA expression data [210, 211], lncRNA expression data [212, 213], methylation data [214, 215], and protein data [216].

Specifically, in proteomics, gene pair methods can be used to analyze the relative relationships of protein expression levels. Proteomics data is based on the abundance of proteins, like gene expression data in transcriptomics. By selecting protein pairs (e.g., protein A and protein B) and calculating their relative expression relationship (e.g., whether protein A is higher than protein B), features can be constructed for classification or state differentiation without relying on absolute expression levels. This relative abundance-based analysis has significant advantages, such as eliminating common batch effects and quantitative biases introduced by different protein measurement methods. Similarly, in metabolomics, gene pair methods can be used to analyze the relative relationships of metabolite abundances. Metabolomics data consists of a large number of small molecules that directly or indirectly reflect the metabolic state of cells [217]. By constructing metabolite pairs and comparing their relative abundances, disease-related metabolic pathways or key biomarkers can be revealed. For example, in metabolic diseases like type 2 diabetes, changes in the relative abundance of certain metabolite pairs can help distinguish disease states. Additionally, this method is robust to batch effects commonly found in metabolomics experiments, further enhancing its applicability in multi-center studies. In epigenomics, the concept of gene pair methods can also be extended to epigenetic data, such as DNA methylation or histone modifications. For example, by comparing the methylation levels of two CpG sites or the modification intensity of two histone modification sites, relative relationships can be established for constructing features in classification models. This analysis helps identify key patterns in epigenetic regulation and reveals epigenetic markers for specific diseases.

Furthermore, with the rapid advancement of single-cell RNA sequencing (scRNA-seq) technologies [218], many gene pair methods based on scRNA-seq data have also emerged. Chen et al. constructed a new feature matrix from scRNA-seq data, known as the “delta rank matrix” (DRM), based on the relative expression order of gene pairs. The results showed that DRM outperformed the original gene expression matrix in cell clustering, cell identification, and pseudotime reconstruction [219]. Tong et al. proposed “scRankXMBD”, a computational framework based on the relative expression order of gene pairs in scRNA-seq data, aimed at prioritizing the identification of prognosis-related cell subpopulations, demonstrating higher accuracy and consistency compared to five existing methods [220]. Shen et al. introduced a novel ensemble learning method named “scDetect” which combines the ranking relationships of gene pairs with a majority voting ensemble learning strategy, enabling high-precision cell classification across scRNA-seq data from different sequencing platforms [221]. Additionally, Yan et al. developed a new method called “RankCompV3”, which can identify differentially expressed genes in scRNA-seq data by comparing the relative expression order of gene pairs [222]. These methods also exhibit significant potential for clinical applications [223].

### Extension to multiclass classification

The methods introduced in this review are primarily based on binary classification problems, but they can also be extended to multiclass classification problems. During this extension, well-known strategies such as “One-vs-One” [224], “One-vs-All” [225], and “Hierarchical Classification” [226] are commonly used. These strategies address multiclass problems by combining multiple binary classifiers.

In the “One-vs-One” method, a binary classifier is trained for each pair of categories. This means that if there are *N* categories, N*(*N*−1)/2 binary classifiers are needed, each comparing two categories, and the final class of each sample is determined through a voting mechanism. The advantage of this method is that it allows for more refined learning of the boundaries between different categories. However, its drawback is that when the number of categories is large, the computational cost increases significantly, as the number of classifiers grows quadratically. In the “One-vs-All” strategy, each category is distinguished from all other categories. This means that for each category, a binary classifier is trained to determine whether a sample belongs to that category. The final classification is made by evaluating the output probabilities of each classifier and selecting the category with the highest probability. Compared to “One-vs-One”, this method is simpler, requiring *N* classifiers. However, when the categories are highly similar, it may face a decrease in classification performance. “Hierarchical classification” addresses multi-class classification problems by constructing a hierarchy of categories. This method typically starts with coarse classification and progressively refines the categorization. The core idea is to gradually narrow down the classification scope using a hierarchical structure, which improves classification accuracy. For example, samples might first be categorized into broad groups (e.g., disease vs. health), and then further subdivided into specific subclasses (e.g., different types of diseases). “Hierarchical classification” can effectively reduce the number of classifiers needed, avoiding the direct distinction between all categories, thus improving both classification efficiency and accuracy [227].

Additionally, Marzouka et al. developed an R package called “multiclassPairs” specifically designed for training multiclass classifiers based on feature pairs [228]. With these extensions, the gene pair methods can not only effectively address binary classification problems but also tackle challenges in multiclass classification tasks.

## Discussion and conclusion

Gene pair methods analyze data by leveraging the combined information of two genes, offering more comprehensive and nuanced biological insights. Notably, these methods are inherently robust to batch effects, making them highly effective for mining diverse biological datasets across different experimental conditions. This review systematically examined eleven distinct gene pair methods, emphasizing their methodologies, applications, and implementation details. By categorizing these methods based on expression values and ranking relationships, we provided a comprehensive overview to guide researchers in their application. Additionally, we developed a reproducible analytical pipeline based on these methods, ensuring their accessibility and usability for practical research scenarios. While most of these methods lack publicly available implementation tools, we have made every effort to reproduce the corresponding code and analyses based on descriptions in published manuscripts. This absence of reference tools posed significant challenges, as relying solely on method descriptions may introduce inconsistencies or biases. Consequently, we cannot guarantee that the reproduced results are fully identical to those in the original studies. This highlights the importance of providing open-source implementations when developing methods, as they not only help other researchers validate and apply these methods but also enhance the credibility and reliability of scientific discoveries.

Beyond summarizing and reproducing these approaches, we evaluated their performance using real-world benchmark datasets (PTB transcriptomic profiles) to highlight their strengths and limitations. These evaluations revealed that gene pair methods are robust tools for extracting meaningful biological insights from high-throughput gene expression data, offering significant advantages in disease research.

In the eleven methods reviewed in this paper, except for RankComp, which is used for the identification of personalized differentially expressed genes, the other ten methods are primarily applied to various classification tasks. Among these methods, three are based on gene expression values, with Pair t-score and PSVM being complex and not capable of eliminating batch effects, whereas GERs, in contrast, can overcome this issue. The remaining seven methods are based on ranking relationships. TSP, although the first method proposed using ranking relationships, relies solely on the top scoring gene pairs for classification. While it uses fewer features, it typically does not achieve the best performance compared to other methods. *k*-TSP, on the other hand, builds on TSP by increasing the number of feature gene pairs used for classification, thus enhancing its discriminatory power. Furthermore, corresponding packages have been developed by researchers to facilitate its application. REOs introduces the concept of reversed gene pairs and, like *k*-TSP, works with multiple gene pairs, achieving similar effectiveness. Additionally, TSPG, while ensuring the stability of identified feature gene pairs through repeated random sampling strategies, also increases the computational burden. Finally, by integrating methods such as TSP, *k*-TSP, and REOs with machine learning, the application performance can be enhanced, although their effectiveness depends on the coding implementation skills of researchers,

In practical applications, based on different research scenarios, we offer the following recommendations: For studies aiming to identify robust differentially expressed genes, we recommend using RankComp, as it has shown good robustness in such analyses. For studies involving multi-batch data integration, methods like Pair t-score and PSVM are not suitable due to their limitations in handling batch effects. We suggest using other methods, such as *k*-TSP, REOs, that are more robust against batch effects. For datasets with a large number of features (e.g., over 20,000), we do not recommend using Pair t-score, as it is computationally expensive. Similarly, methods like TSP and TSP + ML are also time-consuming in such cases and are less ideal. For bench researchers without programming backgrounds, we suggest using methods with well-established toolkits, such as TSP and *k*-TSP, which offer convenient solutions for experimental analysis. Lastly, we encourage researchers to use multiple methods for comparative analysis to identify the most suitable approach for their specific data. We provide the code implementation and comparative analysis of these methods for reference (10.5281/zenodo.14948408).

However, the implementation of gene pair-based methodologies must account for their intrinsic constraints. The reduction of continuous expression data to binary ordinal relationships inherently sacrifices quantitative information about expression magnitudes, which may mask biologically critical subtle variations. Technical artifacts (e.g., measurement noise in low-abundance genes) and threshold-dependent analytical frameworks could additionally undermine cross-platform reproducibility. From a clinical perspective, despite their resistance to batch effects, persistent challenges include tumor microenvironment heterogeneity, dynamically evolving disease states, and limited biological interpretability of mechanistically unrelated gene pairs. Moreover, the absence of standardized clinical validation frameworks demands concerted efforts to bridge the gap between computational research and real-world implementation.

Looking forward, gene pair-based approaches offer substantial potential for advancing precision medicine across a wide range of clinical and research scenarios. These include identifying differentially expressed genes, supporting basic research, facilitating disease diagnosis, distinguishing subtypes, tracking disease progression, and predicting treatment efficacy, thereby providing valuable insights for diverse studies. Furthermore, the application of gene pair methods has expanded to other data types, such as single-cell sequencing, further demonstrating their versatility and broad utility in addressing complex biological questions. These methods not only provide novel perspectives but also serve as powerful tools for both medical research and clinical practice. Future efforts could focus on enhancing computational efficiency, integrating multi-omic data, and improving result interpretability to better support clinical decision-making.

In summary, by summarizing existing methods and providing a reproducible pipeline, this review aims to serve as a valuable resource for researchers, inspiring future innovations and fostering broader applications of gene pair analysis in biomedical research.

## Data Availability

Not applicable.
